# Economic evaluation of infliximab, synthetic triple therapy and methotrexate in the treatment of newly diagnosed juvenile idiopathic arthritis

**DOI:** 10.1186/s12969-022-00748-w

**Published:** 2022-11-16

**Authors:** Maarit Tarkiainen, Pirjo Tynjälä, Paula Vähäsalo, Kristiina Aalto, Liisa Kröger, Katariina Rebane, Pekka Lahdenne, Janne Martikainen

**Affiliations:** 1 New Children’s Hospital, Helsinki University Central Hospital, PO Box 705 00029 HUS, Helsinki, Finland; 2 Pediatric Research Center, University of Helsinki, Helsinki, Finland; 3 University of Helsinki, Helsinki, Finland; 4 PEDEGO Research Unit, University of Oulu, Oulu, Finland; 5 Department of pediatrics, Oulu University Central Hospital, Oulu, Finland; 6 Department of pediatrics, Kuopio University Hospital, Kuopio, Finland; 7 School of Pharmacy, University of Eastern Finland, Kuopio, Finland

**Keywords:** Juvenile idiopathic arthritis, Biological therapy, Disease-modifying anti-rheumatic drugs; health economic evaluation

## Abstract

**Background:**

Evaluation of costs and short-term cost-effectiveness of infliximab plus methotrexate (IFX + MTX); triple therapy of hydroxychloquine, sulphasalazine, and methotrexate (TRIPLE); or methotrexate monotherapy (MTX) in patients with new-onset polyarticular juvenile idiopathic arthritis (JIA).

**Methods:**

In a prospective multicenter study (ACUTE-JIA), costs and health outcomes of 60 randomized patients with new-onset disease-modifying anti-rheumatic drug (DMARD)-naïve polyarticular JIA were analyzed during the first year. A mapping algorithm was used to obtain utility values from Child Health Assessment Questionnaire (CHAQ). Wallace criteriae were used to assess clinically inactive disease (CID). Linear regression with non-parametric bootstrapping was used to adjust imbalances at baseline.

**Results:**

Using prices for IFX biosimilar, adjusted annual mean (SD) costs of treatment (€) were 21,164 (4158), 12,136 (5286), and 18,300 (8635) on IFX + MTX, TRIPLE, and MTX, respectively. Incremental cost-effectiveness ratio (ICER) for IFX + MTX as compared with TRIPLE or MTX were 3442 € or 678 € per additional month spent in CID. Mean (SD) quality-adjusted life years (QALYs) for IFX + MTX, TRIPLE and MTX were 0.755 (0.065), 0.725 (0.062), and 0.686 (0.124). ICER for IFX + MTX vs TRIPLE was 294,433 €, and for IFX + MTX vs MTX 31,435 € per QALY gained.

**Conclusions:**

In short-term, biosimilar IFX + MTX can be considered cost-effective when compared with MTX alone. TRIPLE was cost-effective when compared with MTX and showed cost advantage when compared with IFX + MTX. Cost per time spent in CID showed similar results than ICER evaluations.

**Trial registration:**

This trial was primarily registered with the Ethical Board of Helsinki District University Hospital (https://www.hus.fi), clinical trial number 211864, and later with ClinicalTrials.gov, number NCT01015547.

**Supplementary Information:**

The online version contains supplementary material available at 10.1186/s12969-022-00748-w.

## Background

Juvenile Idiopathic Arthritis (JIA) is an autoimmune rheumatoid condition starting in childhood, with incidence of 7–23/100,000 [1]. In 40–60% of the patients, the disease continues into adulthood [2], possibly causing significant functional impairment and economic burden. The current recommended initial therapy for patients with polyarticular JIA is methotrexate (MTX) monotherapy [3]. Biologics are used as second-line therapy.

For the time being, biologic therapies are much more expensive than conventional disease-modifying anti-rheumatic drugs (DMARDs), although biosimilars have reduced the costs of therapy in some countries. In JIA, medication costs became more than 3-fold when DMARDs were switched to etanercept [4]. In adult rheumatoid arthritis, costs of biologics were 2–3-fold compared with DMARDs, but the estimated addition of quality-adjusted life-years (QALY) was minimal [5, 6].

The original study (ACUTE-JIA) focused on early aggressive therapy in patients with new-onset polyarticular JIA, who were biologic and DMARD naïve. Due to availability of biologics at onset of the study, we chose infliximab, which is widely used off-label in care of JIA. Direct comparisons between different anti-TNF products are missing. However, their efficacy and safety profiles have indirectly shown similarities [7], and thus we find infliximab treatment representative for anti-TNFs as a group. The results of ACUTE-JIA demonstrated superior efficacy of biologic over conventional therapy. Moreover, combination of three DMARDs was more effective than DMARD monotherapy [8]. In polyarticular JIA, biologics in combination with DMARDs have also shown good efficacy and safety profile [9, 10].

In children, only few direct cost-effectiveness comparisons of different therapies of JIA exist [11, 12]. To our knowledge, reports on costs of biosimilar therapies in JIA have not been published to date. In this study, we used the data collected in the ACUTE-JIA study to assess short-term costs and cost-effectiveness of infliximab plus methotrexate compared with triple or monotherapy of DMARDs in an early aggressive treat-to-target approach.

## Methods

The present study was part of the ACUTE-JIA study, a multicenter, randomized, and controlled trial in which 60 patients with new-onset polyarticular JIA were randomized with a blinded block randomization method to receive either infliximab plus methotrexate (IFX + MTX); a triple therapy of hydroxychloroquine, sulphasalazine, and methotrexate (TRIPLE); or methotrexate monotherapy (MTX). Infliximab dose was 3–5 mg/kg and it was administered at weeks 0, 2, 6, and within 6 weeks’ interval thereafter. The initial doses of hydroxychloroquine, sulphasalazine, or methotrexate were 5 mg/kg daily, up to 300 mg; 40 mg/kg daily, up to 2000 mg; or 15 mg/m2/week up to 25 mg, respectively. If the ACRpedi criteriae were not met from week 12 onwards, methotrexate could be doubled to a subcutaneous dose of 30 m2/week, up to 25 mg. Data on the health outcomes and costs were collected during the first year of treatment from eight study visits altogether. A treat-to-target approach was applied, and during the one-year study period the target was set to 75% improvement from baseline. The ACUTE-JIA study protocol has been previously described in detail [8].

### Resource use and costs

The cost-effectiveness analysis considered both healthcare and societal perspectives. The resource use was collected alongside with the ACUTE-JIA study including the use of anti-rheumatic drugs, intravenous infusions, intra-articular injections, primary and tertiary care outpatient visits, laboratory tests, imaging, therapist visits, and hospital admission days related to JIA. Data on resource use were collected from case report forms and medical charts.

All costs were analyzed in intention-to-treat fashion. When, due to inefficacy, patients in TRIPLE or MTX started biologics, costs of this treatment were allocated to the original group.

We included costs of healthcare visits every 3 months, which would be expected without participating in the study. However, additional visits due to disease activity (i.e. intra-articular injections, infusions, or visits due to possible adverse events) were all recorded as they occurred. Visits, laboratory tests, and imaging made solely due to participating in the study were not included in the costs.

Data related to parental absenteeism and travel expenses associated with JIA treatment were collected with questionnaires at each visit. For travel expenses, we estimated the mean distance to both tertiary hospital and primary health care separately. Since the distances varied a lot based on part of the country, we used the average distances for patients in Oulu (Northern Finland) and Helsinki (capital area) for all patients in the study. For unit price we used the travel cost supplement approved by Finnish Tax Administration [13]. We included all costs of visits as described above, excluding travel costs related solely to study protocol.

For medicines, we used mean prices from wholesale price statistics obtained from IQVIA Inc., weighted on number of buys. Data on unit costs of health care visits and procedures were obtained from the statistic provided by the Finnish National Institute for Health and Welfare [14]. For procedures and visits not included there, Helsinki University Central Hospital price catalogue [15] was used.

For intra-articular injections, costs of anesthesia were included as they occurred, including general inhalation anesthesia, if necessary [15]. For parental absenteeism, we used the mean salary per working day, produced by Finnish Statistical Centre [14]. All prices, including medicines, were converted to year 2015 level using the latest available price index of public health expenditure [16]. Since the costs occurred during one-year follow-up, no discounting was performed.

### Health outcomes

Wallace criteriae [17] were used to assess disease activity: Disease was considered inactive if there were no active joints, erythrocyte sedimentation rate was within the normal range, physician and patient/parent visual analogue scale (VAS) scores were zero, and no active uveitis was detected. Duration of clinically inactive disease (CID) was estimated on a weekly basis [8].

As the secondary outcome, we calculated cost per additional quality-adjusted life-year (QALY) gained. QALYs were obtained from Child Health Assessment Questionnaire (CHAQ) figures using mapping algorithms ([18], Table 1), which produce QALYs related to Euro Quality of Life 5-dimension (EQ-5D) utility figures assuming similar relations in activity and utility than in adult patients.

**Table 1:** Baseline characteristics of randomized patients

	IFX (*N*=20)	TRIPLE (*N*=20)	MTX (*N*=20)
Female, n (%)	14 (70)	14 (70)	11 (55)
Age (years) mean (SD)	10.5 (3.2)	8.3 (2.7)	9.6 (3.2)
Age of onset of JIA, years	10.5 (3.1)	8.1 (2.7)	10.0 (3.5)
ANAAb positive, n (%)	9 (47)	7 (35)	6 (30)
ESR, mm/h, mean (SD)	28 (20)	41 (33)	39 (30)
JADAS-10	17.1 (4.4)	20.1 (5.0)	21.9 (5.4)
Active joint count, mean (SD)	18 (10)	17 (10)	18 (12)
Physician VAS, mean (SD)	49 (18)	55 (19)	60 (18)
CHAQ	0.49 (0.6)	0.71 (0.6)	1.07 (0.6)
EQ-5D ^a^			
Roche quadratic	0.73 (0.14)	0.78 (0.16)	0.60 (0.15)
NICE quadratic	0.68 (0.15)	0.62 (0.18)	0.52 (0.18)
Roche linear	0.75 (0.15)	0.69 (0.18)	0.59 (0.17)
Boggs et al. linear	0.66 (0.16)	0.60 (0.18)	0.49 (0.18)

*IFX* Infliximab, *TRIPLE* Combination of hydroxychloroquine, sulphasalazine, and methotrexate, *MTX* Methotrexate monotherapy, *ANAAb* Anti-nuclear antibody, *JADAS-10* Juvenile arthritis disease activity score, *CHAQ* Child Health Assessment Questionnaire, *EQ-5D* EuroQoL-5D questionnaire. *NICE* National Institute for Health and Care Excellence. Values are presented as mean (SD)

^a^Utility values onset are presented as results of different mapping algorithms [18]

NICE quadratic HRQOL = 0.82–0.11*CHAQ-0.07*(CHAQ^2)

Roche quadratic HRQOL = 0.804–0.203*CHAQ-0.045*(CHAQ^2)

Roche linear HRQOL = 0.89–0.28*CHAQ

Boggs et al. linear HRQOL = 0.76–0.28*CHAQ+ 0.05*female

### Statistical and uncertainty analyses

Linear regression was utilized to assess time spent in inactive disease, adjusted for CHAQ at onset, age, and gender. Patients failing their original treatment strategy remained in their intention-to-treat groups for analysis.

To reduce uncertainty around the incremental cost-effectiveness ratio (ICER) estimates, we ran a pairwise linear regression non-parametric bootstrapping with 1000 iterations. Costs were adjusted for age and gender, and due to numeric differences at onset, for CHAQ. Utility values were adjusted for baseline CHAQ. The statistical analyses were performed with SPSS v23.0, STATA 12.1, and Microsoft Office Excel. Different mapping algorithms for converting CHAQ to utility scores were tested, and prudence principle was applied when selecting algorithm. To characterize the impact of sample uncertainty on cost-effectiveness estimates, cost-effectiveness acceptability frontier (CEAF) based on 1000 bootstrap iterations was applied to show the probability that the optimal treatment option is cost-effective at given willingness-to-pay (WTP) per QALY levels [19].

## Results

### Patient characteristics

Altogether 60 patients between June 2003 and October 2006 were randomized in this study. At baseline, patients in IFX + MTX had lower CHAQ (Table 1), and thus costs and utility in the current work were adjusted for baseline CHAQ. No patient in IFX + MTX, 4 in TRIPLE, and 9 in MTX switched treatment strategy during the first year [8]. Of these, 3 patients in TRIPLE and 6 in MTX were started with biologics.

### Costs

IFX + MTX had the highest medication costs, 12,182€ (SD 4246 €) using the unit price of branded molecule, 7204 (SD 2472) in biosimilar prices. Iv-infusion costs were a considerable cost component in this group, whereas costs of intra-articular steroid injections and physiotherapy were smallest. Total costs were highest in IFX + MTX group 21,164 € (SD 4801), and lowest in TRIPLE; 12,288 € (SD 5545), using unit prices of biosimilars. Costs of intra articular steroid injections and biologics were higher in MTX compared with TRIPLE. (Table 2) Detailed resource use is presented in additional file (Additional file 1).

**Table 2:** Resource use, cost-effectiveness and cost of time in inactive disease during first year from onset of therapy in JIA

	IFX	TRIPLE	MTX
**Resource**			
Mean, € (SD)			
Cost of bDMARDs^a^	6781 (2420)	291 (1020)	1200 (4089)
Total medication costs			
Original	12,182 (4246)	1040 (1290)	2230 (2867)
Biosimilar	7204 (2472)	952 (960)	1953 (2517)
Intra-articular injections			
in general anesthesia	2556 (2225)	3745 (2169)	4815 (1985)
in local anesthesia	118 (315)	101 (188)	236 (331)
Intravenous administration	4095 (688)	193 (614)	429 (912)
Health care visits			
Rheumatologist	1754 (534)	1893 (446)	1823 (420)
Ophtalmologist	849 (272)	1050 (344)	1036 (400)
Dentist	379 (589)	170 (327)	454 (530)
General practitioner	66 (113)	58 (108)	75 (156)
Physiotherapy	943 (693)	1331 (1158)	2217 (1226)
Other therapy	177 (205)	265 (287)	468 (409)
Hospital admission days	90 (293)	60 (261)	299 (659)
Gray scale radiography	70 (105)	59 (93)	170 (194)
Magnetic resonance imaging	167 (296)	100 (238)	416 (505)
**Total Health care costs**			
Original infliximab	23,512 (4077)	10,244 (4908)	15,349 (6782)
Biosimilar infliximab	18,533 (3126)	10,093 (4580)	14,963 (6461)
Work loss	1667 (1701)	1363 (1515)	2239 (2660)
Travel Expenses	963 (202)	681 (257)	1008 (358)
**Total costs, societal perspective**			
Original	26,142 (4801)	12,288 (5545)	18,686 (8872)
Biosimilar	21,164 (4158)	12,136 (5286)	18,300 (8635)
Mean Cumulative QALYs^b^	0.758 (0.065)	0.735 (0.062)	0.666 (0.124)
Mean cum time in CID, weeks	24.8 (17.7)	13.3. (14.4)	6.2 (8.9)
**Incremental differences**^**c**^	IFX vs TRIPLE	TRIPLE vs MTX	IFX vs MTX
**Incremental total costs**^**d**^ **(95%CI)**			
Original	12,516 (11,219 to 13,850)	− 6164 (− 8086 to − 4303)	6353 (4585 to 7999)
Biosimilar	8833 (7209 to 9494)	−6164 (− 8086 to − 4303)	2169 (382 to 3754)
**Incremental QALYs** ^**e**^	0.030 (0.014 to 0.045)	0.039 (0.021 to 0.056)	0.069 (0.053 to 0.084)
ICER, €	IFX vs TRIPLE	TRIPLE vs MTX	IFX vs MTX
Original	417,200	Dominant^e^	92,072
Biosimilar	294,433	Dominant^e^	31,435
**Cost per additional month spent in CID, €** ^**e**^			
	IFX vs Triple	TRIPLE vs MTX	IFX vs MTX
Original	5282	Dominant^f^	1765
Biosimilar	3442	Dominant^f^	678

*IFX* Infliximab, *TRIPLE* Combination of hydroxychloroquine, sulphasalazine, and methotrexate, *MTX* Methotrexate monotherapy; *ia* Intra-articular; *iv* Intravenous, *QALY* Quality-adjusted life-year, *ICER* Incremental cost-effectiveness ratio [(Cost A – Cost B)/(QALY A – QALY B)]; CID = clinically inactive disease;

^a^Biosimilar prices used for bDMARDs, when available

^b^Unadjusted; NICE quadratic equation: NICE quadratic HRQOL = 0.82–0.11*CHAQ-0.07*(CHAQ^2)

^c^CI estimates of linear non-parametric regression, bootstrap of 1000 rounds

^d^Adjusted with age, gender, and CHAQ at onset

^e^Adjusted with utility at onset

^f^TRIPLE is less costly and more effective compared with MTX

### Health outcomes

During the one-year follow-up, patients on IFX + MTX, TRIPLE, or MTX spent altogether 24.8 (SD 17.7), 13.3 (14.4), or 6.2 (8.9) weeks in CID, respectively. Adjusted cumulative QALYs were 0.755 (95%CI 0.754 to 0.755) in IFX + MTX, 0.725 (95%CI 0.724 to 0.725) in TRIPLE, and 0.686 (95% CI 0.686 to 0.686) in MTX (Table 2).

### Cost-effectiveness

**Fig. 1:**
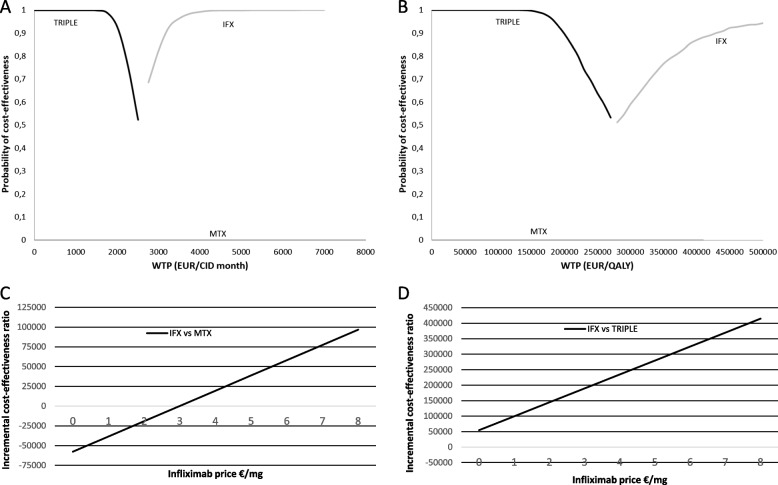
(**A**) Cost-effectiveness acceptability frontier (based on 1000 bootstrap iterations) indicating the probability of the optimal treatment option being cost-effective across different willingness-to-pay (WTP) thresholds per month in clinically inactive disease (CID) and (**B**) per quality-adjusted life-year (QALY) gained. **C** Incremental cost-effectiveness ratio (ICER) of infliximab plus methotrexate (IFX) vs. TRIPLE (methotrexate, hydroxychloroquine, and sulphasalazine) as function of infliximab price. **D** ICER of infliximab plus methotrexate (IFX) vs. methotrexate monotherapy (MTX)

Using biosimilar prices, adjusted cost per additional month spent in CID was 3442 € for IFX + MTX vs TRIPLE, − 3849 € for TRIPLE vs MTX, and 678€ for IFX + MTX vs MTX (Table 2). TRIPLE dominated (i.e. it was less costly and more effective) when it was compared with MTX. A cost-effectiveness acceptability frontier (CEAF) shows that at low levels of willingness-to-pay (WTP) per time spent in CID, TRIPLE is most probably the optimal treatment (Fig. 1A).

In case of QALYs, ICERs of IFX + MTX compared with MTX and TRIPLE were 31,435 €, and 294,433 €, respectively (Table 2). CEAF shows that the probability of TRIPLE being the optimal treatment is high, whereas the probability of IFX + MTX remains low. In terms of cost-effectiveness, MTX has a very low probability of being the optimal treatment (Fig. 1B).

### Sensitivity analysis

When calculating utility, the NICE quadratic algorithm (Table 1) resulted in smallest difference between groups (data not shown), and thus was selected. Evaluation of ICER at different IFX prices shows that IFX + MTX would be cost-effective at current prices, compared with MTX, but not when compared with TRIPLE. (Fig. 1C-D).

## Discussion

In this study, we demonstrated that during the first year of treatment of polyarticular JIA, IFX + MTX had the highest total costs. For IFX + MTX, costs of intravenous administration were a significant cost component. For TRIPLE or MTX, costs of intra articular injections, mainly costs of general anesthesia, were the largest cost component. Almost half of the patients originally in the MTX group did not reach the target and were switched mostly to biologic therapy. In the analyses, costs were calculated as an intention-to-treat fashion. Therefore, in MTX, costs of biologics in patients failing the original strategy were a significant cost component.

When calculating cost-effectiveness for IFX + MTX compared with MTX, incremental cost per additional month spent in inactive disease was less than 1000 €. Results were similar when applying cost per QALYs. ICER of IFX + MTX compared with MTX was within the QALY threshold limit £20,000–30,000 of NICE evaluations [20], indicating acceptable additional cost of a more effective treatment. However, interpretation of these results has to be made with extraordinary caution. The results are applicable only for patients with polyarticular JIA during the first year from disease onset, and in an aggressive treat-to-target setting.

Interestingly, when IFX + MTX was compared with TRIPLE using incremental costs per additional month spent in inactive disease or per QALY, estimates were quite favorable for TRIPLE. Furthermore, when analyzing all three strategies, at any acceptable willingness-to-pay level, TRIPLE seemed most optimal treatment. In adult patients with rheumatoid arthritis, triple conventional DMARD therapy has been shown to be effective and cost-effective [5, 6, 21]. However, European League against Rheumatism (EULAR) and ACR recommendations do not include triple therapy for treatment of early RA or JIA [3, 22].

In children, concerns for side-effects and difficulties with medication adherence of DMARDs in a real-life setting have limited considerably their use as a combination. In the ACUTE-JIA study, only 20% of the patients discontinued TRIPLE during the first year due to inefficacy or adverse effects and were started with biologics. A favorable efficacy and safety profile of conventional DMARDs was also shown in a recent Dutch study, where regardless of initial treatment, sequential DMARD monotherapy, MTX in combination with prednisolone or MTX with etanercept, children with early JIA reached inactive disease equally well [23]. It can be stated that considering cost-effectiveness, efficacy, and satisfactory tolerability, studies of new treatment strategies with synthetic DMARDs are warranted for patients with polyarticular JIA. Especially in circumstances with constrained resources, combination of csDMARDs might be a feasible alternative to bDMARDs.

Costs of JIA treatment vary from 952 Can$ (MTX alone) [11] to 45,227 € (majority of patients on biologics) [24]. These depend on country, healthcare system, and medication used in different studies, which makes comparisons of results challenging. Total costs measured in this study were comparable to those in earlier studies reporting all costs and using biologics for refractory patients [24]. In this study, infusion costs seemed to be a major cost component using IFX. In general, this is a considerable disadvantage for biologics administered intravenously [11].

In the present study, patients received original IFX product. Nowadays, more choices for cost-conscious clinicians are available. In adult rheumatoid arthritis (RA), biosimilars and the original IFX product have shown comparable efficacy [25]. In our analyses, we used prices of biosimilars to enable comparisons of costs between IFX + MTX and other treatments. Furthermore, we analyzed ICER at different IFX prices.

The strength of this study is that information on efficacy, and all costs were carefully collected from the patient records and case report forms and included as they occurred. Intensification of treatment occurred in similar approach than in current treatment guidelines. To our knowledge, this is among the first studies considering cost of time spent in CID. Time spent in CID seems to predict long-term outcome in JIA [26], and thus, cost per time spent in CID could be considered a valid instrument when assessing cost-effectiveness of therapies of JIA.

Infliximab, although not licensed, is widely used off-label in JIA. Considering the similarities in efficacy and adverse events profiles of anti-TNF inhibitors [7], the results of this study can be considered relevant in comparing anti-TNF agents as a group to therapy with DMARDs only. One limitation of this study is the time frame of 1 year. However, previous studies have pointed out the significance of attaining early disease control [27, 28], and therefore, the costs of the treatment during the first year are essential for the future course of the disease. For indirect costs, travel costs and parental work loss were included. Due to the short time frame of the study, costs for patient productivity loss or early retirement could not be calculated. To tackle the limited sample size of this study, cost-effectiveness acceptability frontier assessment based on 1000 non-parametric bootstrap iterations was performed. The rather small sample size did not seem to be an important factor in causing uncertainty.

Health utility impacts were not directly measured in the present study. Therefore, we converted CHAQ to utility values applying several algorithms created for converting adult HAQ values to utility values. Using the NICE quadratic algorithm in this work included assumptions of equality between adult HAQ and CHAQ, or EQ-5D and children’s health-related quality of life. We found the use of adult algorithm justifiable, because the utility values in active adult rheumatoid arthritis have shown similarities to utility values in active JIA [12]. In sensitivity analyses, additional equations were used. Furthermore, the primary endpoint, costs per time spent in CID, demonstrated similar results than the ICER estimates.

During one-year time horizon in patients with polyarticular JIA, both IFX + MTX and combination therapy of DMARDs can be considered cost-effective, when compared with MTX alone. A combination of DMARDs showed cost advantages, when compared with IFX + MTX. To confirm the short-term findings of this study, long-term real-world effectiveness and cost-effectiveness studies with larger number of patients are warranted. Combination of DMARDs should also be evaluated when contrasting biologics with other therapies for JIA. In future, economic evaluations on novel, non-TNF biologic therapies of JIA are also needed.

## Conclusions

IFX + MTX had the highest total costs during the first year of treatment of JIA. However, compared with MTX monotherapy, biosimilar IFX + MTX could be considered cost-effective at the commonly accepted willingness-to-pay level of £30,000/QALY. TRIPLE showed good efficacy at a reasonable cost level, and thus should not be forgotten as a therapeutic option in JIA.

## Supplementary Information

**Additional file 1.** Resource use during the first year treatment of Juvenile Idiopathic Arthritis.

## Data Availability

The data that support the findings of this study are available from the ACUTE-JIA investigators, but restrictions apply to the availability of these data, which were used under license for the current study, and so are not publicly available. Data are however available from the authors upon reasonable request and with permission of the ACUTE-JIA investigators.

## Abbreviations

ACUTE-JIA: Aggressive Combination Drug Therapy in Very Early Polyarticular Juvenile Idiopathic Arthritis
IFX: Infliximab
TRIPLE: Triple therapy of hydroxychloquine, sulphasalazine, and methotrexate
MTX: Methotrexate
JIA: Juvenile idiopathic arthritis
DMARD: Disease-modifying anti-rheumatic drug
CHAQ: Child Health Assessment Questionnaire
CID: Clinically inactive disease
ICER: Incremental cost-effectiveness ratio
QALY: Quality-adjusted life-year
VAS: Visual analogue scale
EQ-5D: Euro Quality of Life 5-Dimension
CEAF: Cost-effectiveness acceptability frontier
WTP: Willingness-to-pay
HAQ: Health Assessment Questionnaire
