# Supplementary material for: Economic evaluation of infliximab, synthetic triple therapy and methotrexate in the treatment of newly diagnosed juvenile idiopathic arthritis
Source: Pediatr Rheumatol Online J. 2022 Nov 16;20:97. doi: 10.1186/s12969-022-00748-w (PMC9670564; doi:10.1186/s12969-022-00748-w)
Supplement: Supplementary file 1 — Additional file 1. Resource use during the first year treatment of Juvenile Idiopathic Arthritis. [file 12969_2022_748_MOESM1_ESM.docx]

**Additional File 1.**

**Resource use during the first year treatment of Juvenile Idiopathic Arthritis.**

**Resource use**

Mean (SD) IFX TRIPLE MTX Unit price (€)

Infliximab, mg 1483 (529) 45 (143) 115 (237) 7.66 ^a^; 4.34^b^

Infliximab n (%) 20 (100) 2 (10) 4 (20)

Etanercept, mg 14 (60) 24 (106) 6.09

Etanercept n (%) 1 (5) 1 (5)

Adalimumab, mg 36.0 (161.0) 14.66

Adalimumab n (%) 1 (5)

MTX oral, mg 894 (263) 648 (343) 604 (364) 0.11

MTX oral n (%) 20 (100) 20 (100) 20 (100)

MTX sub cutaneous 42 (188) 302 (443) 481 (484) 1.07

MTX subcutaneous % 1 (5) 7 (35) 12 (60)

Hydroxychloroquine, g 43 (22) 1.16

Hydroxychloroquine n (%) 20 (100)

Sulphasalazine, g 370 (204) 0.31

Sulphasalazine n (%) 20 (100)

Glucocorticoids oral, mg 92 (283) 292 (675) 0.008

Glucocorticoids oral n (%) 2 (10) 6 (30)

Methylprednisolone ia, mg 243 (299) 463 (348) 478 (362) 0.21

Triamsinolone ia, mg 92 (127) 76 (60) 154 (126) 0.78

Naproxen, g 146 (51) 72 (57) 123 (92) 0.62

Health care visits

Rheumatologist 5.1 (1.5) 5.5 (1.3) 5 (1.2) 347

Ophthalmologist 3.0 (0.9) 3.7 (1.2) 3.6 (1.4) 288

Dentist 1.0 (1.6) 0.5 (0.9) 1.2 (1.4) 378

General practitioner 0.4 (0.7) 0.4 (0.7) 0.5 (0.9) 167

ia injections

general anesthesia 2.2 (1.9) 3.2 (1.8) 4.1 (1.7) 1 189

local anesthesia 0.4 (0.9) 0.3 (0.6) 0.7 (1.0) 377

iv administration 9.6 (1.6) 0.5 (1.4) 1.0 (2.1) 429

Physiotherapy

institution 15.6 (11.4) 22.0 (19.1) 36.6 (20.2) 61

home 0.8 (3.3) 2.0 (8.9) 229

Occupational therapy 0.6 (1.2) 0.6 (1.1) 1.4 (1.9) 69

Other therapy^c^ 2.0 (2.3) 3.0 (3.2) 5.3 (4.6) 88

Hospital admission days 0.2 (0.5) 0.1 (0.4) 0.5 (1.1) 599

Phone calls 1.2 (1.6) 1.4 (2.2) 1.3 (1.6) 28

Grey scale radiography 1.2 (1.8) 1.0 (1.6) 2.9 (3.3) 59

MR examinations 0.5 (0.9) 0.3 (0.7) 1.3 (1.5) 333

Visits to tertiary health care 23.8 (5.1) 15.8 (5.7) 19.9 (8.7)

Visits to primary health care 16.0 (11.5) 22.3 (18.9) 37.0 (19.9)

Days of parental absenteeism 9.3 (9.5) 7.6 (8.5) 13.0 (14.9)

IFX: infliximab; TRIPLE: triple therapy of hydroxychloroquine, sulphasalazine, and methotrexate; MTX: methotrexate monotherapy; ia: intra articular; iv: intravenous; MR: magnetic resonance imaging; ^a^ originator infliximab; ^b^ biosimilar infliximab; ^c^ ergotherapist, social worker, psychologist, or rehabilitation nurse.
